# Complement C1q in plasma induces nonspecific binding of poly(acrylic acid)-coated upconverting nanoparticle antibody conjugates

**DOI:** 10.1007/s00216-022-04021-7

**Published:** 2022-03-24

**Authors:** Saara Kuusinen, Miikka Ekman, Kirsti Raiko, Heidi Hannula, Annika Lyytikäinen, Satu Lahtinen, Tero Soukka

**Affiliations:** Department of Life Technologies, Faculty of Technology, University of Turku, Kiinamyllynkatu 10, 20520 Turku, Finland

**Keywords:** Sandwich immunoassay, Nanoparticles, Matrix effect, Upconversion

## Abstract

Upconverting nanoparticles are attractive reporters for immunoassays, because their high specific activity and lack of autofluorescence background enable their detection at extremely low concentrations. However, the sensitivity achieved with heterogeneous sandwich immunoassays using nanoparticle reporters is generally limited by the nonspecific binding of nanoparticle antibody conjugates to solid supports. In this study, we characterized plasma components associated with elevated nonspecific binding of poly(acrylic acid)-coated upconverting nanoparticles in heterogeneous two-step sandwich immunoassays. Plasma was consecutively fractionated using various chromatographic methods by selecting after each step the fractions producing the highest nonspecific binding of upconverting nanoparticle conjugates in an immunoassay for cardiac troponin I. Finally, the proteins in the fractions associated with highest amount of nonspecific binding were separated by gel electrophoresis and identified with mass spectrometry. The results indicated that complement component C1q was present in the fractions associated with the highest signal from nonspecific binding. The interference was not limited to only poly(acrylic acid)-coated nanoparticles or certain antibody combination, but occurred more generally. The interference was removed by increasing the ionic strength of the assay buffer in the sample incubation step or by adding a negatively charged blocker to bind on positively charged C1q, suggesting that the interaction is mostly electrostatic. Hence, we assume that the interference is likely to affect various negatively charged nanoparticles. The identification of complement component C1q as the major interfering protein allows for more rational design of countermeasures in future immunoassay development utilizing nanoparticle reporters.

**Graphical abstract:**

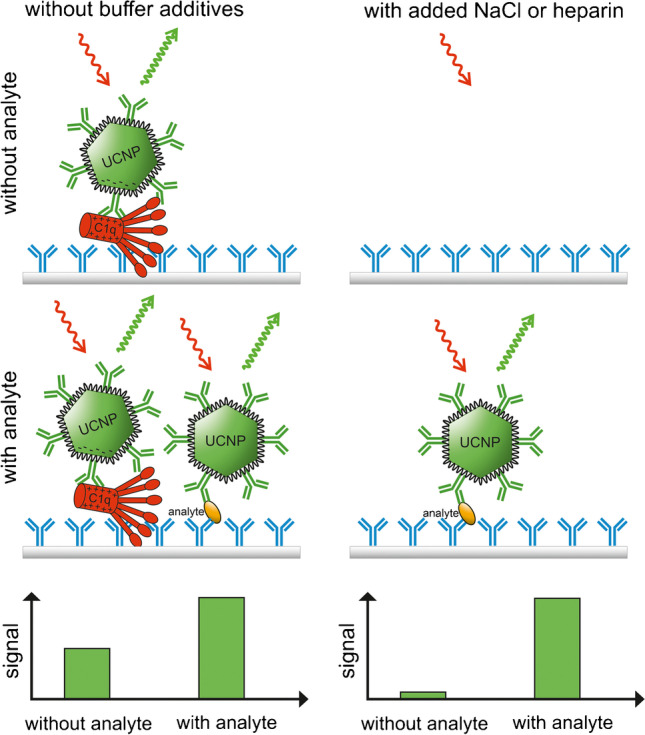

**Supplementary Information:**

The online version contains supplementary material available at 10.1007/s00216-022-04021-7.

## Introduction

Immunoassays are an indispensable tool in diagnostic testing due to their versatility and capability to measure minute concentrations of clinically relevant biomolecules in complex biological matrices. They are commonly carried out in a two-site noncompetitive sandwich immunoassay format, in which two antibodies need to bind to different epitopes of the analyte simultaneously, in order to generate the signal, making the assays highly specific. With optimal antibodies, the sensitivity of this type of assays is generally high, but still limited by either the detectability of the label or by the variation of the elevated background originating from the nonspecific binding of the label antibody conjugates [1].

In such diseases the diagnosis of which requires extremely high sensitivity of analyte detection, the reporter used in the assay has to be detectable at extremely low concentrations. Upconverting nanoparticles (UCNP) have a high specific activity [2], i.e., a high rate of emitted photons per nanoparticle, and a unique ability to convert low-energy excitation light into higher-energy emission. Furthermore, upconversion luminescence (UCL) does not occur in any natural compounds or commonly used solid-phase materials, such as polystyrene or nitrocellulose, enabling luminescence detection without any autofluorescence background [3]. As a result, even single UCNP reporters can be detected [4, 5]. The applicability of UCNPs as labeling reagents in immunoassays has been demonstrated in several studies [4, 6, 7]. The performance of the published assays is at least as good as that of the commercial state-of-the-art assays. However, the sensitivity is still limited by the nonspecific binding of the UCNP antibody conjugates to solid supports, and resolving the issue further could facilitate the development of immunoassays with outstanding sensitivity approaching the nucleic acid amplification–based molecular assays. With nanoparticle reporters, the amount of nonspecific binding is generally higher with biological sample matrices, such as plasma and serum, compared to artificial buffers [7, 8]. This matrix effect can often be decreased by diluting the sample, but the performance of the assay is not necessarily increased as also the analyte concentration decreases with dilution.

The surface chemistry has been concluded to have a considerable effect on the nonspecific interactions of the nanoparticle reporters in biological fluids [9–12]. Nanoparticles have been shown to adsorb proteins from the surrounding solution forming a protein corona on the particle surface, which affects their interactions especially in one-step assays, in which the nanoparticle conjugates are incubated together with the sample. The surface chemistry of nanoparticles has a considerable effect on the formation and composition of protein corona and thus also on the nonspecific binding [13]. Nsubuga et al. [11] observed almost no adsorption of serum proteins on polyethylene glycol (PEG)- and alendronate-coated UCNPs. However, even with PEG-coated UCNPs, nonspecific binding is still limiting the sensitivity of immunoassays [4, 14, 15]. In two-step assays, the nanoparticle conjugates are not in direct contact with the sample, but the nonspecific binding still occurs [4, 15]. Instead of direct adsorption on the nanoparticle surface, the sample components must bind onto the capture surface, to interfere in the following tracer incubation step. The effect of the protein adsorption onto the capture surface in a solid-phase immunoassay, however, has not been studied as extensively as the adsorption on the nanoparticles.

In this study, the plasma proteins associated with the nonspecific binding of poly(acrylic acid) (PAA)-coated UCNPs in heterogeneous sandwich-type immunoassays were investigated, along with the possible mechanism for the interference. A two-step assay format was used, in which the UCNP antibody conjugates are added to the reaction wells in a separate step after the sample incubation and subsequent wash steps. Thus, the interfering substances in the sample matrix must first bind to the surface of the solid phase coated with capture antibodies, strongly enough to not be detached in the washing step, and then interact with the UCNP antibody conjugates. The aim of this study was to enrich and identify the substances in plasma associated with the nonspecific binding, and to design specific ways to block that interaction, and thus, enable more sensitive immunoassays. In order to do that, plasma was fractionated by various consecutive precipitative and chromatographic methods, and after each step, the fractions producing the highest nonspecific binding of UCNP conjugates in a cardiac troponin I (cTnI) immunoassay were combined for further purification. Finally, the proteins present in the interfering fractions were separated using gel electrophoresis and identified with mass spectrometry (MS).

## Experimental section

### Materials

Blood for the plasma pool was collected in lithium heparin vacuum tubes (Vacuette® 9 mL, Greiner Bio-one, Kremsmünster, Austria) from 10 apparently healthy volunteers from Southwest Finland in compliance with the declaration of Helsinki. Before pooling, the samples were anonymized and the plasma was separated by centrifugation according to the instructions provided by the manufacturer. The UV-Star microtiter plates for the absorbance measurements were purchased from Greiner Bio-One. The Nunc C8 Lockwell LUMI White Maxisorp microtitration plates were purchased from Thermo Fisher Scientific (Waltham, MA, USA) and coated with streptavidin (IBA, Göttingen, Germany) according to a previously described protocol [16]. Monoclonal antibody (Mab) clones 625 and 19C7 specific for human cTnI were purchased from HyTest (Turku, Finland), and recombinant antigen-binding fragment (Fab) 9707 specific for human cTnI was produced, purified, and biotinylated according to a published protocol [17]. The Mab clones 5409 and 5404 specific for thyroid-stimulating hormone (TSH) were purchased from Medix Biochemica (Espoo, Finland). Goat anti-complement C1q antibody was purchased from Genway Biotech (San Diego, CA, USA), and goat anti-IgM antibody was purchased from Sigma-Aldrich (St. Louis, MO, USA). Anti-C1q and anti-IgM antibodies were labeled with 50-fold molar excess of Tb^3+^-chelate of N^1^-(p-isothiocyanatobenzyl)diethylenetriamine-N^1^, N^2^, N^3^, N^3^-tetraacetic acid (University of Turku, Finland) according to a published protocol [18]. Bovine serum albumin (BSA) was purchased from Bioreba (Reinach, Switzerland). The Mab clones 19C7 and 5404 and BSA were biotinylated according to a previously described protocol [18] using a 15-fold, 20-fold, and 35-fold molar excess of biotin-isothiocyanate, respectively, in pH 9.8 for Mabs and pH 9.3 for BSA. The cTnI analyte was purchased as troponin I-T-C-complex (human heart tissue–derived) from HyTest and the TSH analyte from Scripps Laboratories (San Diego, CA, USA). The assay buffer and wash solution were purchased from Kaivogen (Turku, Finland). Native mouse IgG was purchased from Meridian Life Science (Saco, ME, USA) and denatured mouse IgG was prepared from native mouse IgG by incubating in 63 °C for 30 min. Nonfat bovine milk powder was purchased from Valio (Helsinki, Finland). DELFIA Enhancement Solution (DES) and DELFIA Enhancer were purchased from PerkinElmer (Waltham, MA, USA).

N-(3-dimethylaminopropyl)-N’-ethylcarbodiimide (EDC) and N-hydroxisulfosuccinimide (sulfo-NHS) were purchased from Sigma-Aldrich. Oleic acid capped NaYF_4_:Yb^3+^, Er^3+^ UCNPs with an average diameter of 23.7 nm were synthesized according to a previously published protocol [19]. After the synthesis, the oleic acid was removed and the UCNPs were coated with PAA (average MW 2000, Sigma-Aldrich) as described previously [7]. PAA-coated UCNPs were further conjugated to Mab clones 625 and 5409 and streptavidin with 1 mg of biomolecule per 24, 12, and 5 mg of UCNPs, respectively. The conjugation was done using EDC/sulfo-NHS chemistry as described previously [6]. The reactions were stopped and the surface was blocked by adding 2 M glycine (pH 11) to a final concentration of 50 mM, except for the Mab-625-UCNPs, in which 2-amino-N,N-dimethylacetamide was used instead of glycine. UCNPs (core diameter 32.5 nm) with a 13.7 nm silica coating (obtained from Kaivogen) were conjugated to Mab clone 625 and blocked with glycine.

The Laemmli sample buffer, TGS buffer, Mini-PROTEAN TGX Precast gels, and Precision Plus Protein™ Unstained Protein Standards used in the SDS-PAGE were purchased from Bio-Rad (Hercules, CA, USA). The Coomassie Blue R250 was purchased from Sigma-Aldrich.

### cTnI immunoassay

The cTnI immunoassay was carried out as described earlier [6]. The Mab-625-UCNPs were diluted in modified assay buffer (assay buffer supplemented with 0.2% (w/v) milk powder, 0.8 g∙L^−1^ native mouse IgG, and 0.05 g∙L^−1^ denatured mouse IgG) 30 min before starting the assay. The cTnI calibrators were prepared by diluting human I-T-C-complex in Tris-BSA buffer prepared from Tris buffer (50 mmol∙L^−1^ Tris, pH 7.75, 9 g∙L^−1^ NaCl, and 0.5 g∙L^−1^ NaN_3_) by supplementing with 75 g∙L^−1^ BSA. The streptavidin-coated microtiter plates were prewashed once with wash solution before starting the assay. Biotinylated capture antibodies (150 ng of Mab 19C7 and 50 ng of Fab 9707) were added to each well in 50 µL of assay buffer, and incubated for 30 min in slow shaking. The wells were washed once and 10 µL of sample or calibrator was added to each well with 40 µL of Tris buffer and incubated and washed as before. A total of 200 ng/well of Mab-625-UCNPs were added in 50 µL of modified assay buffer. The UCNP dilutions were sonicated for three 0.5 s pulses with 100% amplitude using VialTweeter sonicator (Hielscher Ultrasonics, Germany) just before adding to the wells. The UCNPs were incubated for 15 min in slow shaking; after which, the wells were washed four times and left to dry for 1 h. The upconversion luminescence at 540 nm was measured with a modified Plate Chameleon fluorometer (Hidex Oy, Finland) equipped with a 980 nm laser [20].

The effect of sample dilution on the nonspecific binding of UCNPs in cTnI immunoassay was studied by varying the ratio of plasma pool and Tris buffer in the total reaction volume of the sample incubation step. Plasma pool was used in 0%, 1%, 5%, 10%, 20%, 50%, 80%, and 100% proportions of the total volume.

### Separation and identification of the plasma proteins associated with nonspecific binding of the UCNPs

The entire separation process is illustrated in SI figure S1. The proteins in the plasma pool were first fractionated based on their solubility by gradually increasing (NH_4_)_2_SO_4_ concentration (10%, 20%, 30%, 40%, and 50% saturated (NH_4_)_2_SO_4_ in 50 mM Tris–HCl (pH 7.75)). The solution was shaken for 25 min, followed by centrifugation at 15,000 g for 20 min at 4 °C; after which, the precipitate was dissolved in the original plasma volume of Tris buffer. The total protein concentration in each fraction was estimated based on absorbance at 280 nm measured with Hidex Sense microplate reader (Hidex Oy). The fractions were tested for nonspecific binding of UCNPs in a heterogeneous cTnI immunoassay.

The fractions with the highest signal from the nonspecific binding were pooled and the components were separated further based on their size. The separation was done by gel filtration chromatography using HiLoad 16/600 Superdex 200 pg column (GE Healthcare, Chicago, IL, USA) with a bed volume of 120 mL, equilibrated and run with Tris buffer (50 mmol∙L^−1^ Tris, pH 7.75, 9 g∙L^−1^ NaCl, and 0.5 g∙L^−1^ NaN_3_) with a flow rate of 1 mL∙min^−1^. The optical density of the elution at 280 nm was monitored with Spectra System UV2000 (Thermo Fisher Scientific). Fractions of 1 mL were collected and analyzed for nonspecific interactions as described before, with the exception that in the sample incubation step, 50 µL of the fractions was added to the wells without any additional Tris buffer. The fractions with the highest signal from the nonspecific binding were pooled, diluted 1:4 with 20 mM Tris–HCl, pH 8.0, and separated based on their charge with anion exchange chromatography (Mono Q 5/50 GL column with a bed volume of 1 mL, GE Healthcare) equilibrated and run with 20 mM Tris–HCl, pH 8.0 with a flow rate of 1 mL∙min^−1^. The proteins were eluted from the column by increasing the NaCl concentration linearly (0.1 M∙min^−1^) from 0 to 1 M with 20 mM Tris–HCl, pH 8.0, 1 M NaCl. The optical density was monitored as before and 250 µL fractions were collected and analyzed as described earlier. In order to prevent the high concentration of NaCl in the anion exchange fractions affecting the level of nonspecific binding in the cTnI immunoassay, the fractions were added to the wells with 20 mM Tris–HCl, pH 8.0, instead of Tris buffer.

Proteins in the purified plasma were separated with SDS-PAGE (Mini-PROTEAN 3 Cell, Bio-Rad). Some of the fractions collected from anion exchange chromatography were diluted with 20 mM Tris–HCl, pH 8.0 due to the high protein concentration, to ensure distinct separation of protein bands. The samples were mixed with equal volume of Laemmli sample buffer or Laemmli sample buffer with 5% β-mercaptoethanol, and denatured in 95 °C for 5 min. The samples were added to the wells of Mini-PROTEAN®﻿ TGX Precast gel in 30 µL volume. Electrophoresis was carried out in TGS buffer (25 mM Tris, 192 mM glycine, 0.1% SDS, pH 8.3) and run under a constant voltage of 100 V for 90 min. The molecular weights of the proteins were determined using Precision Plus Protein™ Unstained Protein Standards. The gel was stained with Coomassie Blue by incubating it in staining solution (0.1% Coomassie Blue R250, 30% methanol, 5% acetic acid) for 1 h and destained with a solution containing 30% methanol and 5% acetic acid. Selected protein lanes were cut from the gel and analyzed with LC–ESI–MS/MS. The MS analysis was purchased from Turku Proteomics Facility (Turku, Finland).

### Characterization of the nonspecific binding

To study whether the interference is generic or limited only to the cTnI immunoassay or PAA-coated UCNPs, the plasma fractions precipitated with < 50% (NH_4_)_2_SO_4_ were pooled and analyzed, in addition to the cTnI immunoassay, also with a TSH immunoassay and by using streptavidin-coated microtiter plate and UCNP-streptavidin conjugates, according to a previously described protocol [21]. The fractions were diluted 1:1 with Tris buffer. Biotinylated BSA (40 ng·mL^−1^ in Tris-BSA buffer) was used as a positive control sample for UCNP-streptavidin conjugates. The nonspecific binding was also cross-tested with capture surface specific to different analytes than the UCNP conjugates to ensure that the observed signal did not originate from endogenous cTnI or TSH present in the plasma pool. The TSH immunoassay was performed as the cTnI immunoassay with some modifications: 100 ng of biotinylated Mab 5404 was added to each well in 50 µL of assay buffer. After 30 min incubation, the wells were washed once and the samples were added, incubated, and washed as in cTnI immunoassay. To each well, 200 ng of Mab-5409-UCNPs were added in 50 µL of modified assay buffer. The UCNP antibody conjugates were incubated and washed and the upconversion luminescence was measured as in cTnI immunoassay. The nonspecific binding was tested with both PAA- and silica-coated UCNPs.

Based on the MS analysis, the complement C1q was found to be present in the plasma fractions associated with a high nonspecific binding in cTnI immunoassay. Hence, the binding of C1q on the solid-phase was determined by adding 100 ng of goat anti-C1q-Tb-N1 conjugate in 50 µL of modified assay buffer to the microtiter wells after the sample incubation step of the cTnI immunoassay. The tracer antibodies were incubated in 600 rpm shaking for 30 min; after which, the wells were washed four times and 150 µL of DES was added to the wells to dissociate the Tb^3+^ ions. The wells were incubated for 15 min in slow shaking, followed by the addition of 30 µL of DELFIA Enhancer to form fluorescent Tb^3+^ chelates. The wells were incubated for 15 min as before and time-resolved fluorescence (TRF) was measured with Victor 1420 multilabel counter (PerkinElmer) using 340 nm excitation filter, 545 nm emission filter, 500 µs delay, and 1400 µs measurement window. Because immunoglobulin M subunits were also present in many of the plasma fractions with high nonspecific binding, the nonspecific binding of IgM was also determined similarly, by using anti-IgM-Tb-N1 conjugate instead of anti-C1q-Tb-N1.

The effect of ionic strength and heparin to the C1q-mediated nonspecific binding was studied by adding 100, 300, or 500 mM NaCl or 50 U∙mL^−1^ heparin to the Tris buffer in the sample incubation step of cTnI immunoassay. Pooled highest nonspecific signal exhibiting anion exchange fractions were diluted 1:1 with Tris buffer and used as samples. A similar experiment was repeated with anti-C1q-Tb-N1 as described before, to study whether the effect in nonspecific binding due to additions of NaCl and heparin correlates with their effects to C1q binding onto the capture surface.

## Results and discussion

**Fig. 1:**
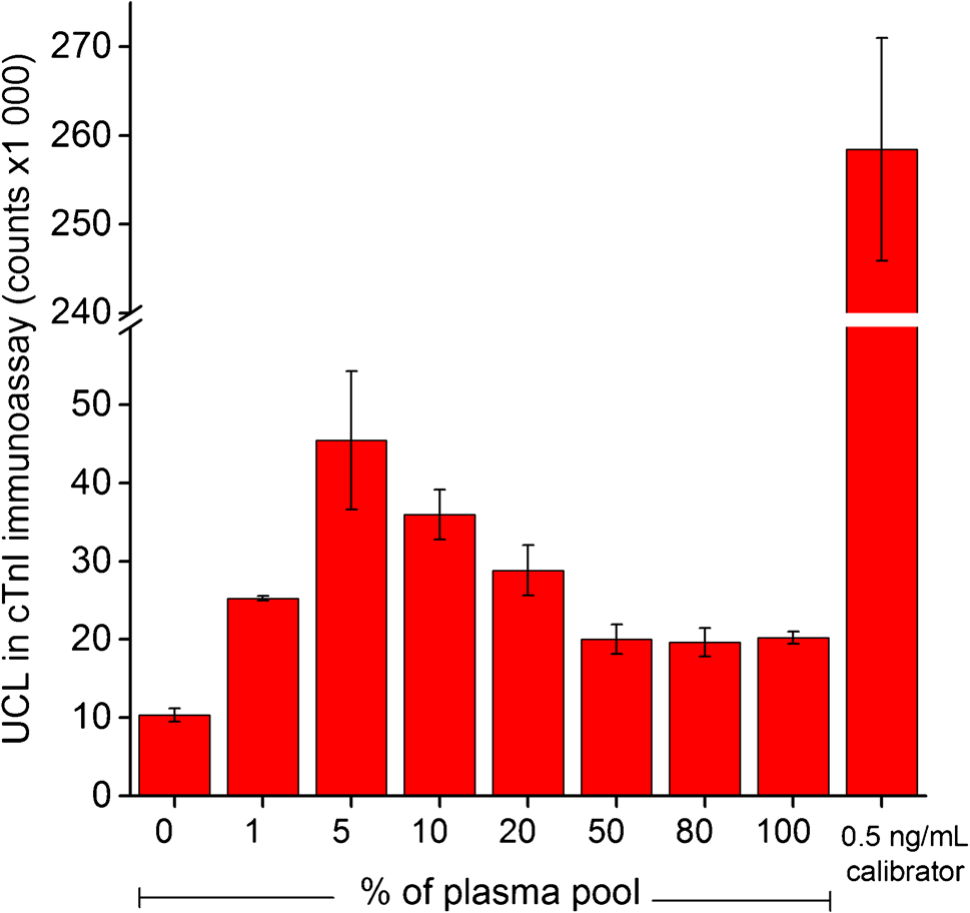
Effect of plasma pool dilution on nonspecific binding of UCNPs. Lithium heparin plasma pool was diluted in Tris buffer and analyzed with cTnI immunoassay. 0.5 ng∙mL^−1^ of cTnI was spiked into Tris-BSA buffer and analyzed using 10% dilution

**Fig. 2:**
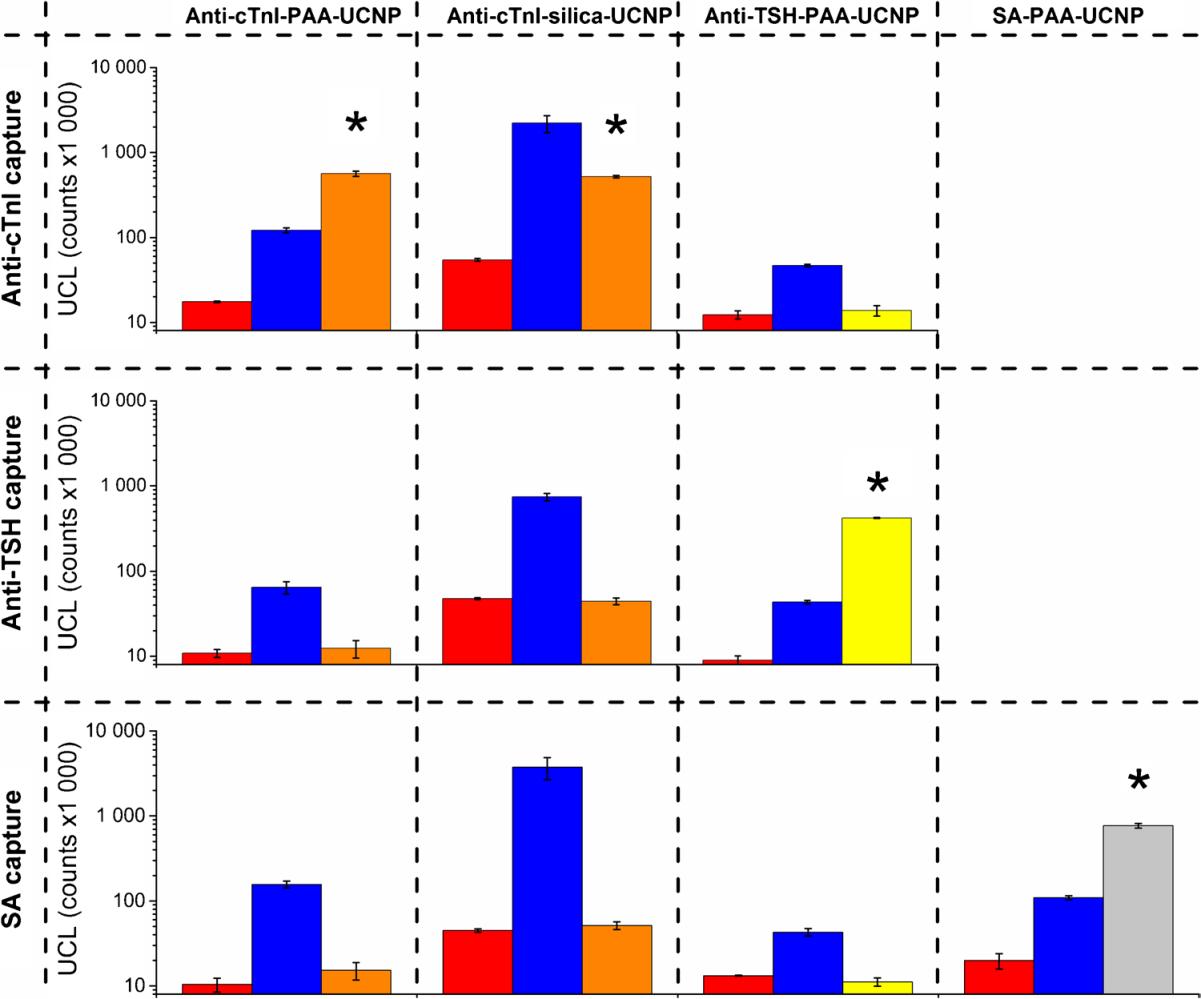
Cross-testing of immunoassay capture surfaces and tracer conjugates. UCNP tracer conjugates of cTnI and TSH immunoassays and biotin-BSA binding assay were tested with anti-cTnI, anti-TSH, and streptavidin capture surfaces. Red bars represent nonspecific binding with Tris-BSA buffer as a sample, and blue bars with plasma fractions precipitated with < 50% (NH_4_)_2_SO_4_ as a sample. Orange, yellow, and grey bars represent binding with Tris-BSA buffer spiked with 0.5 ng∙µL^−1^ of cTnI, 2.5 ng∙mL^−1^ of TSH, or 40 ng∙mL^−1^ of biotin-BSA, respectively. The combinations resulting in specific binding via analyte molecules are marked with an asterisk

Diluting the plasma pool with Tris buffer surprisingly resulted in increased nonspecific binding in cTnI immunoassay compared to that of undiluted plasma (Fig. 1). Typically, the matrix effects in immunoassays are decreased when the samples are diluted, but here, the effect of dilution was the opposite. This hypothesis-opposing finding could have been a result of matrix effects blocking the detection of the analyte. However, elevated signals of nonspecific binding were also obtained from (NH_4_)_2_SO_4_ precipitated plasma fractions by using a crosswise antibody combination not specific to any analyte (Fig. 2). The signal was thus attributed to be caused by the nonspecific binding of UCNPs to components present in the plasma pool. The signal of the nonspecific binding was increased with higher dilution down to a 5% proportion of plasma. The phenomenon was likely caused by co-diluting also the substances blocking the nonspecific interactions in the heparin plasma pool. As the substances with blocking effects were diluted, the interfering substances could interact more strongly with UCNPs, causing the increase in nonspecific binding even though their concentrations were also diluted. However, with any analyzed plasma proportion, the nonspecific binding was approximately 100–350% higher than with Tris buffer alone.

**Fig. 3:**
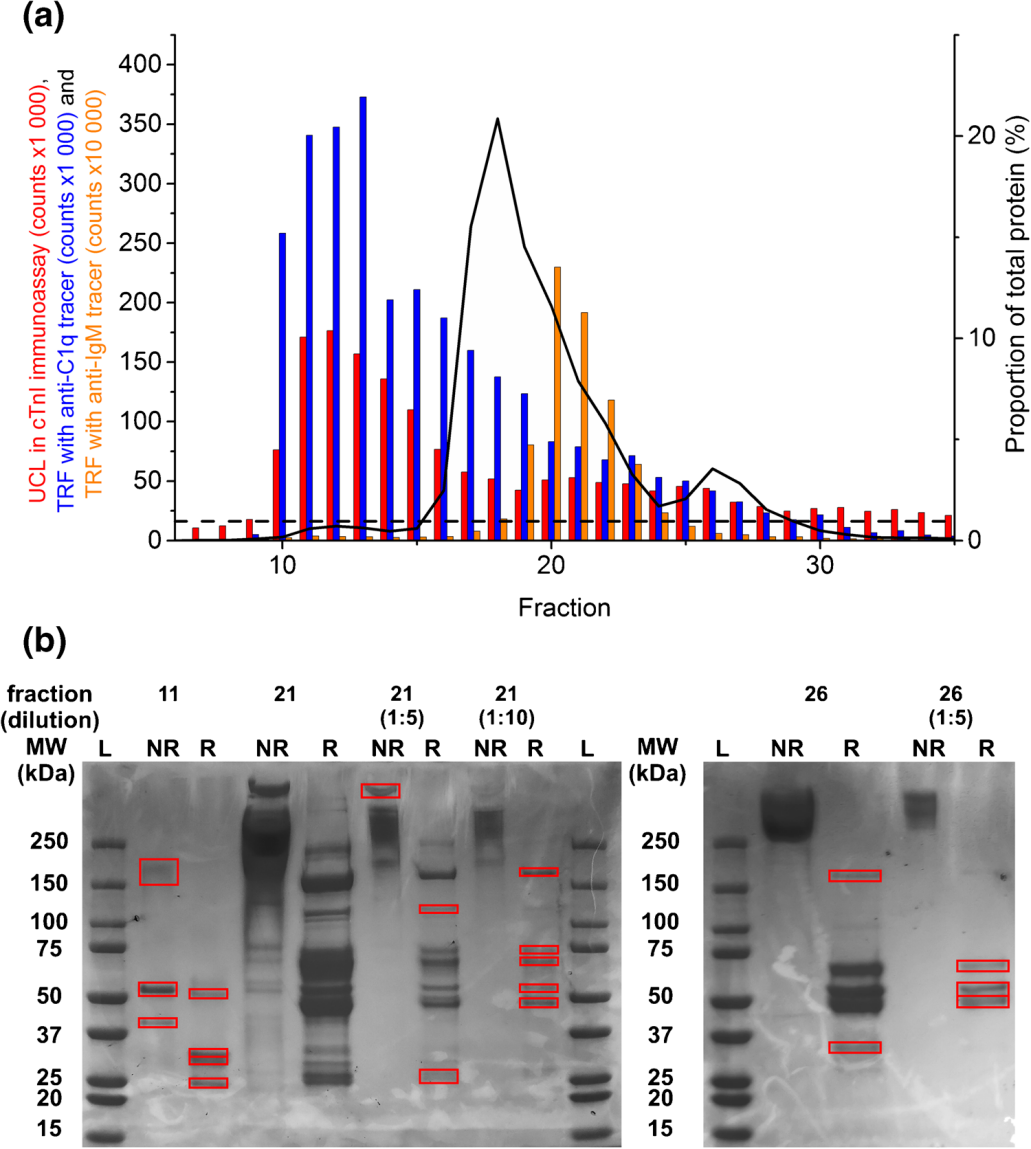
**a** UCL signals in cTnI immunoassay of anion exchange chromatography fractions (red bars), fluorescence signals with polyclonal anti-C1q (blue bars), and with anti-IgM (orange bars) tracers. The total protein concentrations in the plasma fractions (black line) were estimated based on absorbance at 280 nm. The dashed horizontal line shows the level of nonspecific binding of UCNP antibody conjugates with Tris-BSA buffer. **b** SDS-PAGE analysis of purified plasma fractions. The fractions were analyzed in nonreduced (NR) and reduced (R) form with 5% β-mercaptoethanol. The protein bands analyzed with MS are highlighted with red boxes. Molecular weight standards are marked with L

The analysis results of (NH_4_)_2_SO_4_ precipitated and gel chromatography separated plasma fractions, and the fractions pooled for the next purification step are presented in SI figures S2 and S3. Anion exchange chromatography resulted in three distinct peaks of elevated nonspecific binding in cTnI immunoassay (Fig. 3a ). In anion exchange chromatography, the proteins with the most positive net charge elute first, followed by the proteins with neutral and negative charge. The highest nonspecific binding was measured in the first peak (fractions 10–16), in which the total protein concentration was relatively low, suggesting that most of the nonspecific binding was caused by a small, positively charged proportion of the total protein in plasma. The UCL signal in the second peak of nonspecific binding was lower than in the first peak and it was located around fraction 21, where the total protein concentration was approximately tenfold compared to the first peak. The third peak of nonspecific binding (fractions 25–26) was the lowest, but still approximately twice as high as the nonspecific binding with Tris-BSA buffer as a blank sample.

From each of the three peaks, one fraction associated with high nonspecific binding was analyzed with SDS-PAGE (Fig. 3b) and selected protein bands were identified with MS. The MS results showed that complement component C1q was present in all of the plasma fractions analyzed with SDS-PAGE. A full summary of the MS results is provided in the supporting data files.

C1q is a part of the C1 complex in the classical complement pathway of the immune system and consists of six triple-helical strands, each of which contains three different polypeptide chains (A = 28 kDa, B = 25 kDa, and C = 24 kDa) [24]. With an isoelectric point (pI) of 9.3, it is one of the most positively charged proteins in human plasma [22]. Chains A and B are linked together from their N-terminal halves via disulfide bonds. Chain C is disulfide-linked to the chain C of the next triple-helical strand, binding the strands together from their N-terminal halves and forming a bouquet-like hexameric structure [23, 24]. Each of the strands has a C-terminal globular head with binding sites for several ligands, such as IgG, IgM, and molecular patterns on microorganisms [24–28]. The molecular weights of the chains A, B, and C match the three protein lanes on the reduced form of fraction 6 on the SDS-PAGE. The A-B and C–C dimers have molecular weights of approximately 54 and 48 kDa, respectively, which match the protein band close to 50 kDa in the nonreduced form of fraction 6 (Fig. 3b).

The plasma fractions with the highest nonspecific binding of UCNPs also resulted in the highest fluorescence signals when detected with anti-C1q-Tb-N1 tracer conjugate, confirming that C1q had bound to the microtiter well surface and was not removed by the subsequent washing of the wells (Fig. 3a). In the second peak of nonspecific binding, the fluorescence signal from the anti-C1q-Tb-N1 tracer conjugate was approximately 80% lower than in the first peak, while the UCL signal from the nonspecific binding decreased by 70%, indicating that some other proteins may also contribute to the nonspecific binding. It is also possible that C1q is present as complexes with other proteins, like IgM (pI 4–9.1 [29]), causing part of C1q to elute later, and the complexes may have different tendencies to cause nonspecific binding. Thus, even IgM was observed in the second peak of nonspecific binding (anion exchange fractions 19–24), it is not necessarily itself responsible for the nonspecific binding, but may be present due to complexation to C1q. Thus, the nonspecific binding of the UCNP antibody conjugates in plasma seems to be mostly dependent on the presence of C1q. Anti-C1q and anti-IgM antibodies were labeled with molecular terbium chelate labels instead of nanoparticle reporters to avoid measuring nanoparticle-associated nonspecific binding with them. It is likely that the anti-C1q antibody conjugates truly measure the amount of C1q bound to the surface and the binding is not nonspecific, because the anti-C1q antibodies bound to different anion exchange chromatography fractions than the anti-IgM antibodies.

To show that the nonspecific binding is not limited to a certain antibody combination and to ensure that the purification did not result in enrichment of endogenous cTnI, capture surfaces and tracer conjugates of cTnI immunoassay and TSH immunoassay, as well as UCNP-streptavidin conjugates and streptavidin-coated surface, were tested crosswise. With all capture-tracer combinations, the nonspecific binding with pooled (NH_4_)_2_SO_4_ precipitated (< 50%) plasma fractions was consistently higher than with Tris-BSA buffer (Fig. 2), confirming that the purification did not enrich endogenous cTnI. The plasma-associated increase in the UCL was discovered also with silica-coated UCNPs, showing that the nonspecific binding is not specific only for PAA-coated UCNPs. Human C1q has been shown to have an affinity towards mouse IgG antibodies [30], which are widely used in immunoassays and were also used in the immunoassays in this study. The affinity of C1q towards mouse IgG antibodies could obviously cause bridging of capture and tracer antibodies without the presence of the analyte, thus leading to nonspecific binding of UCNP conjugates. However, nonspecific binding occurred even in the biotin-BSA binding assay with the streptavidin-coated surface. Therefore, antibodies are not needed for nonspecific binding.

As an alternative to the antibody-mediated binding, the interaction could also be electrostatic. Gorris et al. [31] discovered a strong correlation between the positive net charge of peptides and nonspecific binding onto an antibody-coated polystyrene surface. The strong positive charge of C1q could also lead to an ionic interaction with negatively charged nanoparticles. Tavano et al. [32] demonstrated that C1q can bind to poly(2-methyl-2-oxazoline)-coated silica nanoparticles without the presence of IgG and IgM antibodies. This could also occur with the PAA- and silica-coated UCNPs, as all of these nanoparticles have a negatively charged surface coating.

**Fig. 4:**
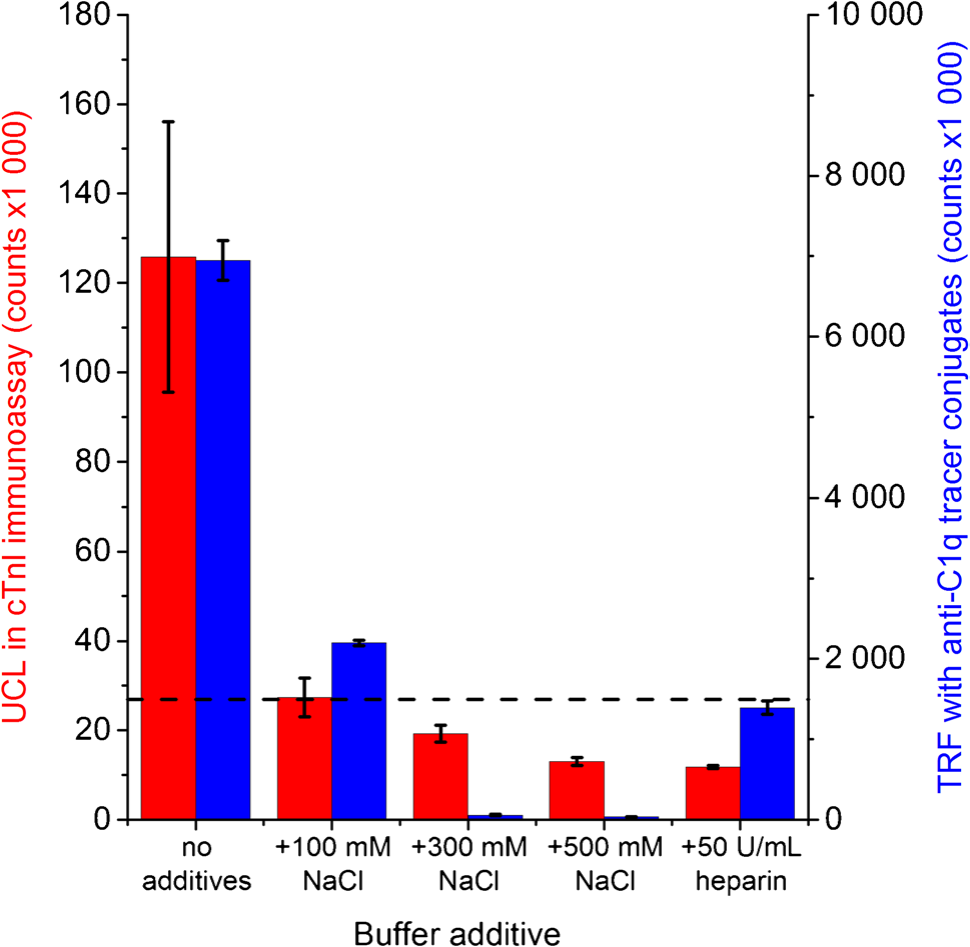
Effect of buffer additives on the nonspecific binding of UCNPs in cTnI immunoassay (red) and fluorescence signal with anti-C1q tracer conjugates (blue). Anion exchange fractions associated with the highest nonspecific binding were pooled and analyzed with cTnI immunoassay and with anti-C1q-Tb-N1 conjugates. The additions were made into Tris buffer comprising ca. 150 mM NaCl in the sample incubation step. The dashed line represents the nonspecific UCL with Tris-BSA buffer as a sample matrix

The binding of antibodies to the globular heads of C1q is considered mainly electrostatic and thus sensitive to ionic strength [33–35]. Kaul et al. [35] showed that the binding of C1q to an antibody-coated surface can be prevented by adding NaCl at the same time with C1q. Additionally, if the high positive net charge of C1q causes nonspecific binding of negatively charged UCNP conjugates, it should be prevented by increasing the ionic strength or adding a negatively charged blocker. Therefore, the effects of ionic strength and polyanionic blocker on the nonspecific binding of UCNPs were tested by adding 100, 300, or 500 mM of NaCl or 50 U∙mL^−1^ of heparin to the assay buffer in the sample incubation step of cTnI immunoassay with anion exchange fractions associated with high background. The binding of C1q onto the capture surface was also tested with anti-C1q tracer conjugates. The results show that already 100 mM NaCl addition, i.e., the total concentration of ca. 250 mM NaCl, efficiently decreases nonspecific binding of UCNPs, resulting in UCL signals similar to those of nonspecific binding in Tris-BSA buffer (Fig. 4). The signal measured with anti-C1q tracer conjugates decreased by approximately 68%, indicating that the increased ionic strength also reduced the binding of C1q onto the capture surface. The nonspecific binding of UCNPs was further decreased by increasing the NaCl concentration to 500 mM, which also lead to an almost complete loss of signal with anti-C1q tracer conjugates. Heparin also effectively prevented the nonspecific binding of UCNPs and decreased the fluorescence signal measured with anti-C1q tracer conjugates by approximately 80%. Moreover, the added NaCl (+ 500 mM) or heparin (+ 50 U∙mL^−1^) resulted in a 20% and 47% increase, respectively, in specific UCL signals with cTnI calibrators (data not shown).

The observation that strongly negatively charged heparin effectively decreases the nonspecific binding supports the conclusion that the mechanism of the interaction is mainly electrostatic. This also explains why lithium heparin plasma samples result in a generally lower background compared to that of EDTA plasma samples (SI Figure S5). Additionally, the dilution of heparin could also cause the increase in the nonspecific binding with diluted lithium heparin plasma samples (Fig. 1). Juntunen et al. [36] discovered that, also in lateral flow immunoassays, EDTA plasma as a sample matrix leads to aggregation and nonspecific binding of nanoparticle antibody conjugates, whereas lithium heparin plasma did not have a similar effect. Lateral flow immunoassays are done in one-step format, and therefore, the nanoparticle conjugates are in direct contact with the sample material. It is possible that the aggregation of nanoparticle conjugates was caused by C1q in plasma. Since heparin decreases the nonspecific binding of UCNPs, it probably also prevents their aggregation in plasma.

The observation that both, NaCl and heparin, prevented the binding of C1q and the nonspecific binding of UCNPs to the surface supports the conclusion that C1q causes the plasma-associated nonspecific binding and that the binding can be prevented by blocking the interactions of C1q.

## Conclusions

According to this study, the complement component C1q is likely responsible for a major part of the plasma-associated nonspecific binding of PAA-coated UCNP antibody conjugates in heterogeneous immunoassays. The gel filtration and anion exchange chromatography lead to a distinction between the highest peak in the nonspecific binding of UCNPs and the main protein peak, suggesting that the enrichment of the components associated with nonspecific binding was successful.

The immunoassays were carried out in a two-step format, in which the UCNP conjugates were not mixed with the sample but incubated in a separate step after washing. Thus, C1q must first bind to the capture surface to be available for interaction with UCNPs. Previously, it has been shown that C1q binds to IgG-coated surfaces strongly enough to not be detached even with high ionic strength wash buffer [35]. In this study, the nonspecific binding was not limited to antibody-coated surfaces, but was observed also on surfaces coated with streptavidin, which rules out the interaction mechanism only based on the ability of C1q to bind antibodies. This supports the conclusion of mainly electrostatic interaction.

It is likely that the results of this study are not limited only to PAA-coated UCNPs, but apply also for other negatively charged nanoparticle reporters. Although a negative charge improves the colloidal stability and monodispersity of nanoparticles in aqueous solutions, it also makes them vulnerable to charge-dependent interactions in biological matrices. Therefore, a weaker negative charge could be beneficial for nanoparticles that are used in diagnostic applications, and careful optimization of the surface chemistry of the nanoparticles is crucial in the development of ultrasensitive assays [37]. The nonspecific binding can also be prevented by additives blocking the electrostatic interactions, like NaCl and heparin, but their applicability depends on the target analyte and the antibodies used, and suitable methods need to be determined specifically for each assay. In the future, a similar strategy could be used to enrich and identify sample compounds associated with nonspecific binding of different types of labels or label conjugates.

## Supplementary Information

Below is the link to the electronic supplementary material.

Supplementary file1 (DOCX 134 KB)

Supplementary file2 (XLSX 622 KB)

## Declarations

### Human plasma samples

All participants gave their informed consent for participation.
