# Supplementary material for: Complement C1q in plasma induces nonspecific binding of poly(acrylic acid)-coated upconverting nanoparticle antibody conjugates
Source: Anal Bioanal Chem. 2022 Mar 24;414(12):3741–9. doi: 10.1007/s00216-022-04021-7 (PMC9035425; doi:10.1007/s00216-022-04021-7)
Supplement: Supplementary file 1 — Supplementary file1 (DOCX 134 KB) [file 216_2022_4021_MOESM1_ESM.docx]

Electronic Supporting Information

Complement C1q in plasma induces nonspecific binding of poly(acrylic acid) coated upconverting nanoparticle antibody conjugates

Saara Kuusinen*, Miikka Ekman, Kirsti Raiko, Heidi Hannula, Annika Lyytikäinen, Satu Lahtinen & Tero Soukka

Department of Life Technologies, Faculty of Technology, University of Turku, Kiinamyllynkatu 10, FI-20520 Turku

*Corresponding author: [saevku@utu.fi](mailto:saevku@utu.fi)

Contents

[1. Fractionation of the plasma components and analysis of the nonspecific binding associated with the fractions 1](#_Toc94199012)

[2. Effect of sample matrix in cTnI immunoassay 5](#_Toc94199013)

# Fractionation of the plasma components and analysis of the nonspecific binding associated with the fractions

The fractionation process is presented as a flow chart in figure 1. The interfering compounds were enriched from the plasma pool of healthy donors by (NH_4_)_2_SO_4_ precipitation, gel filtration chromatography and anion exchange chromatography. After each purification step, the fractions were analyzed for nonspecific binding of UCNPs in a cTnI immunoassay and the protein concentrations were determined based on absorbance at 280 nm. The fractions associated with highest nonspecific binding of UCNPs were pooled for the next purification step. The results of the analysis after each purification step are presented in figures 2–4.

**
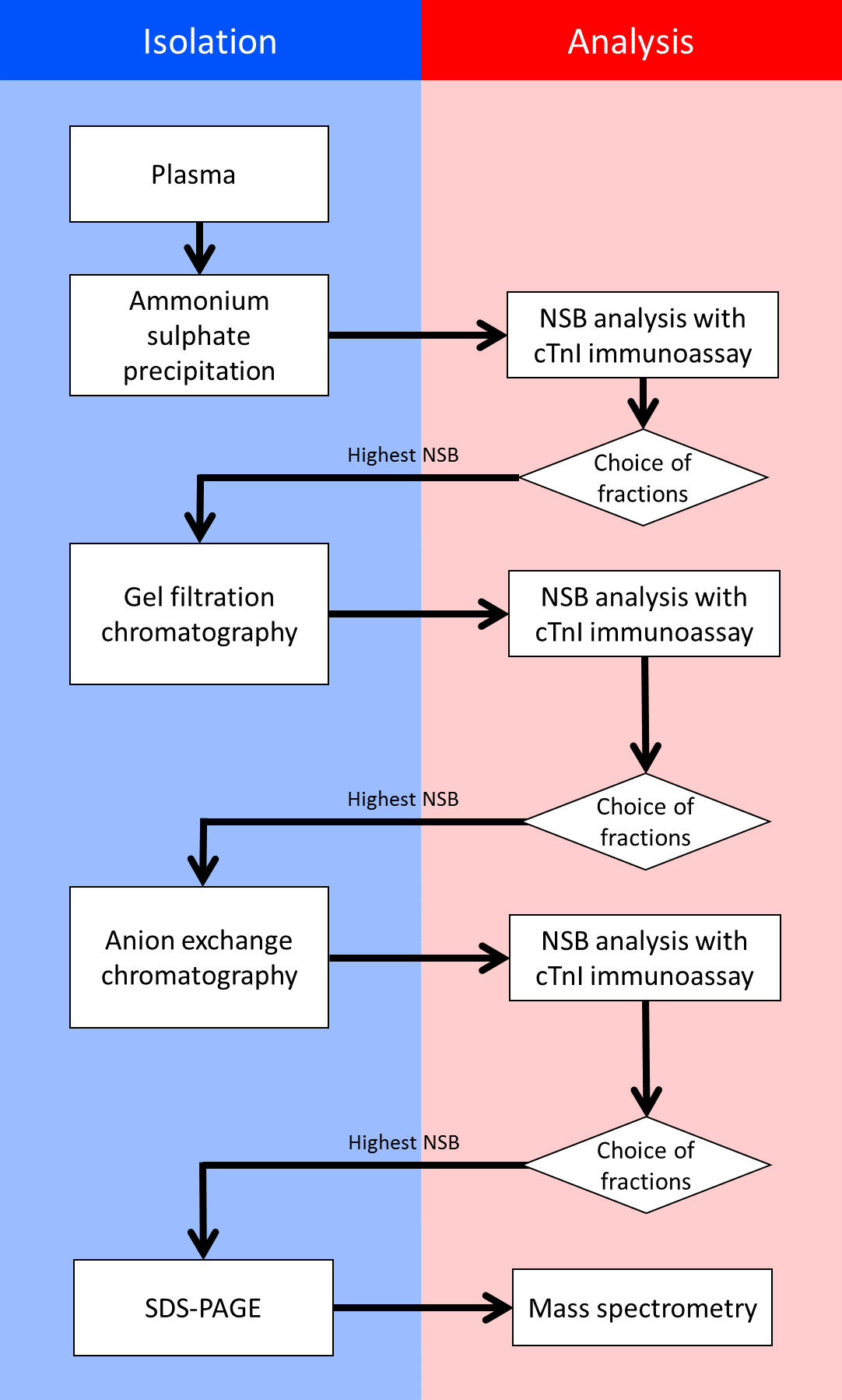
**

***Figure S1.*** *Flow chart of the fractionation of the plasma components and enrichment of the components associated with the nonspecific binding (NSB) of UCNP antibody conjugates.*

Fractions precipitated with 20 and 30% saturated (NH_4_)_2_SO_4_ contained less than 17% of total protein content, but exhibited significantly higher nonspecific binding compared to other fractions (Figure 2). Those fractions were pooled for further purification with gel filtration chromatography.

**Figure S2.** UCL signals in cTnI immunoassay (red bars) and proportion of the total protein concentration based on absorbance at 280 nm (black line) of (NH_4_)_2_SO_4_ precipitation fractions

Highnonspecific binding was observed with gel filtration fractions 15–34, which were clearly separate from the fractions 36-44 with highest protein content (Figure 3). The fractions 21–30 were pooled and further fractionated with anion exchange chromatography.

**Figure S3.** UCL signals in cTnI immunoassay (red bars) and proportion of the total protein concentration based on absorbance at 280 nm (solid line) of gel filtration chromatography fractions. The dashed line shows the nonspecific UCL signal level with blank buffer calibrator.

The anion exchange chromatography resulted in three separate peaks of nonspecific binding, the first of which was the highest (Figure 4). The fractions with elevated non-specific binding were clearly separate from the fractions with the highest protein concentration. One fraction from each peak (fractions 11, 21 and 26) was analyzed with SDS-PAGE and MS. The MS results of selected protein bands are presented in a separate Excel file.

**Figure S4.** UCL signals in cTnI immunoassay (red) and proportion of the total protein concentration (based on absorbance at 280 nm, solid line) of anion exchange chromatography fractions**.** The dashed line shows the nonspecific UCL signal level with blank buffer calibrator. Fractions marked with asterisk were analyzed with SDS-PAGE and MS.

# Effect of sample matrix in cTnI immunoassay

Tris-BSA buffer, as well as pooled EDTA and lithium heparin plasma from healthy donors were spiked with cTnI (0, 0.05, 0.5 and 5 ng∙mL^-1^) and analyzed in a cTnI immunoassay. With EDTA plasma as a sample matrix, the signal originating from the nonspecific binding was more than 200-fold higher than with lithium heparin plasma and almost 400-fold higher than with Tris-BSA buffer (Figure 5).

**Figure S5.** UCL in cTnI immunoassay with Tris-BSA buffer (black), lithium heparin plasma pool (red) and EDTA plasma pool (blue) as a sample matrix.
